# The role of stress echocardiography in identifying cardiotoxicity: an in-depth exploration

**DOI:** 10.3389/fcvm.2024.1236966

**Published:** 2024-01-26

**Authors:** Sijia Wang, Yi Wang, Shuang Wang

**Affiliations:** ^1^ Ultrasound in Cardiac Electrophysiology and Biomechanics Key Laboratory of Sichuan Province, Sichuan Provincial People's Hospital, School of Medicine, University of Electronic Science and Technology of China, Chengdu, China; ^2^ Department of Geriatrics, The People's Hospital of Changshou, Chongqing, China

**Keywords:** stress echocardiography, cardiotoxicity, chemotherapy, left ventricular ejection fraction, speckle tracking echocardiography

## Abstract

Cancer treatment might cause heart failure and deteriorate the patients’ quality of life. Despite the wide use of conventional echocardiography, it often fails to detect cardiotoxicity until advanced cardiac dysfunction at potentially irreversible stages. Advanced techniques, such as three-dimensional imaging and strain analysis in stress echocardiography, have shown promise in identifying cardiotoxicity at subclinical stages, even when traditional measures remain within normal ranges. These novel techniques have been shown to identify cardiac impairment in 30%–50% of the patients undergoing potentially cardiotoxic chemotherapy, which allows for early intervention and enhanced patient management. Although professional societies are advocating for the inclusion of these techniques into routine monitoring protocols, more research is needed to optimize and standardize their use across various centers and chemotherapeutic agents. This review explores the role of stress echocardiography in the early detection and monitoring of chemotherapy-induced cardiotoxicity. It delves into current knowledge and emerging research, aiming to provide a comprehensive understanding and to highlight areas worthy of further investigation.

## 1 Introduction

Advancements in cancer treatment have significantly improved the survival rates, such that the cancer survivors in the United States reached over 5.4% of the population as of January 2022 (1). However, potent antineoplastic therapies, including anthracyclines, taxanes, and targeted agents, have been linked to acute and chronic cardiotoxic effects, which would increase morbidity and mortality (2).

Cardiotoxicity is a detrimental outcome of chemotherapy, which is marked by a decrease in the cardiac function during or after treatment. This represents an alarming condition due to its associated risks of heart failure and subsequent impact on the patient's quality of life (3). Consequently, there is a critical need for effective tools for the early detection, monitoring and prediction of cardiotoxicity. Traditional diagnosis and monitoring of cardiac dysfunction have been heavily based on conventional echocardiography, which quantifies the functional and structural parameters, such as the Left Ventricular Ejection Fraction (LVEF), Fractional Shortening (FS), diameters and volumes. However, these conventional measurements often only allow for the diagnosis of cardiac dysfunction at a late stage, at which point the damage may already be irreversible (4, 5).

Stress echocardiography (SE) employing advanced techniques, such as three-dimensional imaging and strain analysis, has emerged as a powerful tool to detect the signs of cardiotoxicity at subclinical stages when conventional measures, such as ejection fraction, may remain preserved (6). Numerous studies have demonstrated the abilities of these advanced echocardiographic modalities to detect subtle changes in myocardial contractility and deformation even when no abnormalities are recorded with standard metrics, thereby identifying cardiac impairment in 30%–50% of the patients receiving potentially cardiotoxic chemotherapy (7, 8).

Professional societies, including the American Society of Echocardiography and the European Association of Cardiovascular Imaging, advocate for incorporating advanced stress echocardiographic parameters into the monitoring protocols for patients receiving chemotherapy with risk factors. This approach enables earlier diagnosis and management of cardiotoxicity before reaching the stage of irreversible cardiomyopathy (9). The advancements in functional cardiac imaging techniques have the potential to reduce heart failure rates and significantly impact long-term survival and quality of life after cancer treatment (10). However, how to optimize and standardize the use of these modalities across centers and for various chemotherapeutic agents is still unclear.

This review critically analyzes the role of stress echocardiography in detecting cardiotoxicity to provide a comprehensive overview of the current understanding of this topic and identify areas in need for further research.

## 2 Cardiotoxicity and its impact on cardiovascular health

### 2.1 Characteristics of cardiotoxicity

Cardiotoxicity refers to the damage to the heart muscle caused by external factors, such as chemotherapy agents, radiation therapy or other medications. In particular, cancer treatments employ agents that effectively target tumor cells, but also pose risks to cardiac cells and the surrounding structures supporting the heart function (11). Cardiotoxic agents can damage critical structures within cardiomyocytes, including DNA, mitochondria and proteins that are involved in contraction and calcium handling, which impairs the myocardial function. This initially asymptomatic damage can progress to symptomatic heart failure in some patients. The median survival in the case of heart failure resulting from chemotherapy is only 9–12 months (12, 13). Even subclinical dysfunction may reduce the long-term cardiovascular reserve and longevity.

Chemotherapeutic agents, such as doxorubicin and other anthracyclines, function through DNA intercalation, inhibition of topoisomerase II (essential for DNA transcription and replication) and generation of iron-based free radicals that damage the surrounding tissue while at the same time killing cancer cells. These actions induce apoptosis and the death of heart muscle cells, infiltrating and disrupting the contractile units of the heart responsible for effective pumping. Furthermore, anthracyclines damage the mitochondria, the energy producers of cells, as well as the sarcoplasmic reticulum, which plays a crucial role in calcium handling during each heartbeat. Destructing these essential structures impairs the ability of the heart ability to contract and respond to physiological stress (14–16). Other chemotherapy agents, such as trastuzumab, pose risks through promoting inflammation, interfering with signaling pathways that are integral to cardiac cell function, and accelerating the development of coronary atherosclerosis. Novel immunotherapies operate by manipulating the immune response of the body. Meanwhile, immune activation and excess production of cytokines can also cause inflammation and damage of the heart (17, 18). Vascular changes may initially induce high blood pressure, thus adding further stress to the heart muscle. Over time, the combination of these mechanisms can induce morphological changes that are detectable through imaging as markers of injury and predictors of functional decline, if identified before the resting functions start to deteriorate (19–21). Additional risk factors for developing cardiotoxicity include pre-existing heart disease and cardiovascular risk factors, including diabetes or obesity, old age and higher cumulative doses or more aggressive treatment regimens (22, 23). Maximizing survival for the patients facing life-threatening cancer often requires intensive therapies that push the limits of normal tissue tolerance. Previous studies (24, 25) have shown that anthracyclines cause dose-dependent cardiotoxicity in up to 36% of patients. The rates of heart failure were found to be 9% at 1 year, 13% at 5 years and 15% at 10 years in patients treated with anthracyclines. As for the monoclonal antibody trastuzumab, it also causes asymptomatic LVEF decline in up to 50% of breast cancer patients and clinically significant heart failure in 2%–4% as a monotherapy, but up to 25.5% when combined with anthracyclines, which necessitates close monitoring (26, 27). Targeted cancer therapies, like tyrosine kinase inhibitors (TKIs), have also been associated with higher risks of cardiotoxicity. A study (28) analyzed 77 randomized trials and found TKIs to be associated with a 1.41-fold higher risk of all-grade cardiovascular events compared with controls, while high-grade (severe) cardiovascular risks were 1.30-fold higher with VEGFR TKIs. Table 1 summarizes the cardiac dysfunction associated with different cancer therapies and their corresponding pathophysiological mechanisms.

**Table 1:** Cardiac dysfunction in different cancer treatment.

Agent	Cardiac dysfunction
Anthracyclines (e.g., Doxorubicin)	LV systolic dysfunction, cardiomyopathy, arrhythmias
Alkylating agents (e.g., Cyclophosphamide)	Minimal toxicity, LV systolic dysfunction, myocarditis
Antimetabolites (e.g., 5-Fluorouracil)	Ischemic heart disease, angina
Taxanes (e.g., Paclitaxel)	Bradycardia, heart blocks
Monoclonal antibodies (e.g., Trastuzumab)	HF, LV systolic dysfunction, hypertension
Tyrosine kinase inhibitors (e.g., Imatinib)	LV dysfunction, HF, hypertension
Immunotherapy (e.g., PD-1/PD-L1 inhibitors)	Myocarditis, LV systolic dysfunction, arrhythmias
Targeted therapy (e.g., Tyrosine kinase inhibitors)	Hypertension, LV systolic dysfunction, HF
Hormone therapy	Hypertension, thromboembolism
Radiation therapy	Pericarditis, coronary artery disease, valvular disease

ROS, reactive oxygen species; LV, left ventricular; HF, heart failure.

### 2.2 Detection of cardiotoxicity

The diagnosis of cardiotoxicity involves a comprehensive and structured evaluation to identify and manage any cardiovascular complications resulting from treatment. This evaluation consists of several crucial steps. Initially, patients are stratified based on standard cardiovascular risk factors such as age, hypertension, smoking, diabetes, and other factors that may predispose an individual to cardiac complications. This stratification ensures that individuals at higher risk receive more intensive monitoring and intervention. Next, a resting 12-lead electrocardiography is performed, with a specific focus on measuring the QT interval. This interval, representing the duration required for the heart muscle to contract and recover, is a vital indicator of potential cardiac issues. Following this, the left ventricular (LV) function is monitored to detect early signs of cardiotoxicity. This is crucial as the left ventricle is responsible for pumping oxygenated blood throughout the body, thereby enabling timely intervention (29–31).

Due to its broad availability, safety, cost-effectiveness and capacity for repeated measurements, echocardiography is the main modality for monitoring cardiac function during chemotherapy. The left ventricular ejection fraction (LVEF) is typically used to estimate the cardiac contractility and define anticancer therapy-related cardiac dysfunction (CTRCD) as a drop of >10% to below the normal value (9, 32). Nevertheless, LVEF is not sensitive enough to detect minor changes in contractility and may not accurately reflect the structural damage until the impairment becomes moderate or severe (5).

The limitations of LVEF are addressed by the speckle-tracking echocardiography (STE), 2D or 3D. It provides an objective measure of myocardial deformation, which enables the detection of subclinical changes in the cardiac function. Emerging research suggests that reduced global longitudinal strain (GLS) can predict cardiotoxicity, heart failure and adverse events in oncology patients (33). Trastuzumab has been reported to achieve significant reductions in GLS in breast cancer patients, either in combination with anthracyclines or as a standalone treatment. These reductions at 3- and 6-months post-treatment initiation underscore the superior prognostic value of GLS over LVEF in this context (4, 7). The ASE/EACVI consensus suggests that a relative decrease of GLS by >15% from baseline represents clinically relevant evidence of subclinical left ventricular dysfunction (34). However, the use of strain imaging requires specialized software and expertise, which limits widespread adoption, and normal ranges still vary between platforms (35).

The evaluation of myocardial function at rest can often be insufficient to diagnose or detect impairment (36, 37). Stress tests can assess the functional responses to exertion or pharmacological stress, revealing regional ischemia that resolves at rest. This capability is provided along with imaging by stress echocardiography, which allows the visualization of wall motion abnormalities during stress, signaling injury or reduced reserve capacity. Recent studies suggest that even with normal LVEF and imaging at rest, stress echocardiography exhibits high sensitivity for revealing cardiotoxicity. An abnormal response to stress can predict the outcome, thus supporting risk stratification and treatment planning (4).

Additional imaging techniques, such as radioisotope ventriculography Multigated Acquisition (MUGA) and cardiac magnetic resonance imaging (MRI), provide further information on the cardiac structure and function. However, due to radiation exposure with MUGA and the high cost and limited availability of MRI, these modalities are not typically used as first-line examinations within the routine monitoring of patients undergoing cardiotoxic therapy (38).

### 2.3 Stress echocardiography to detect cardiotoxicity

In stress echocardiography, the patients have to walk or jog on a treadmill to increase their heart rate and blood pressure, thereby increasing myocardial oxygen consumption to create a stressful condition for the heart. Ultrasound images are collected at rest and peak exercise to (CAD) assess changes in the heart wall and valves during stress. These changes between the rest status and peak exercise may indicate coronary artery disease or other cardiac abnormalities. On the other hand, pharmacologic stress echocardiography uses medications, such as intravenously administered dobutamine, to directly stimulate the heart, thereby increasing the heart rate, contractility and cardiac workload. Like in exercise stress echocardiography, ultrasound images are obtained at rest and during peak stress, and any changes would indicate potential issues for follow-up testing. Pharmacologic stress echocardiography is often utilized for patients who are unable to adequately exercise for the test (39–43). Stress echocardiography holds comparable overall accuracy in cardiotoxicity detection to other techniques, including Single Photon Emission Computed Tomography (SPECT) or MUGA scanning (44). However, stress echocardiography has the benefits of the lower cost, safety and lack of radiation exposure, which makes it particularly suitable, especially for higher risk patients with moderate to high likelihood of cardiotoxicity.

Stress echocardiography can mainly aid in cardiotoxicity detection through the following ways: (1) Detecting a decline in LVEF: A drop in LVEF of 10% or more can indicate cardiotoxicity. Even with normal LVEF at rest, stress echocardiography can reveal declines in LVEF. Civelli et al. (39) used stress echocardiography to detect decreasing LVEF in chemotherapy patients, even when LVEF at rest was still over 55%. Early detection of declining LVEF is crucial for adjusting the treatment before long-term damage occurs. (2) Identifying wall motion abnormalities: Stress echocardiography can detect abnormal movement of the heart wall with high sensitivity, indicating cardiotoxicity even with normal LVEF. Another study found abnormal wall motion in nearly 20% of patients receiving anthracycline chemotherapy despite preserved LVEF. Hence, wall motion abnormalities often precede major LVEF declines, which allows for earlier intervention (45). (3) Monitoring high-risk patients: Frequent stress echocardiograms in patients on high doses of cardiotoxic medications or with pre-existing heart disease offer close monitoring for changes that could indicate cardiotoxicity. A study by Jarfelt M et al. (46) found that adult acute lymphoblastic leukemia survivors treated with anthracyclines showed subclinical cardiac dysfunction at a median of 21 years post-treatment identified through stress echocardiography. In this asymptomatic group, the ejection fraction at peak exercise was significantly lower in anthracycline-exposed survivors compared with healthy controls, the relevant studies of stress echocardiography in cardio oncology as shown in Table 2.

**Table 2:** Studies of stress echocardiography in cardio oncology.

Study	Sample size	Application	Strengths	Limitations	Outcomes
Civelli et al. (39)	49	Early detection of chemo-induced cardiac dysfunction	Serial DSE assessments, exercise stress	Small sample, limited to high-dose chemo, no long-term follow-up	↓ LVEF, ↓ contractile reserve
Hamada et al. (47)	26	Anthracycline cardiotoxicity (High-dose dobutamine stress echo)	Detailed functional assessments	Small sample, no control group	↓ LVEF, ↓ wall thickening
Jarfelt et al. (46)	35	Assessing cardiac function in survivors of childhood leukemia	Long-term follow-up, homogenous cohort	Small sample size, submaximal stress focus	Significant differences in systolic function during maximal exercise
Khouri et al. (48)	57	Cardiac function in breast cancer patients (Doxorubicin)	Advanced 3D echo & GLS	Small sample, no control for stress test	↓ LVEF, ↑ GLS, ↓ Stroke volume
Ryerson et al. (49)	80	Subclinical cardiac dysfunction in anthracycline-treated cancer survivors	Stratified risk groups, thorough assessments	Small sample, selection bias	↑ Resting heart rates, diastolic dysfunction
von Scheidt et al. (50)	127	LV strain in cancer survivors (Submaximal semisupine bicycle exercise)	Submaximal bicycle exercise stress echo	Small sample, restricted to submaximal exercise	↑ GLS with stress

LVEF, left ventricular ejection fraction; GLS, global longitudinal strain; DSE, dobutamine stress echocardiography; Echo, echocardiography; LV, left ventricular; SD, standard deviation; CS, circumferential strain.

While these studies are pioneering in applying SE to detect cardiotoxicity, they often lack robust methodologies capable of distinguishing between cardiotoxicity-induced impairment and other cardiac conditions, such as CAD or diastolic dysfunction. For instance, observed declines in LVEF in several studies may result from poor contractile reserve or CAD. However, current methodologies inadequately differentiate between these scenarios, underscoring the need for future research that incorporates control groups with known CAD or employs additional diagnostic tools like biomarkers to accurately determine the causes of LVEF decline.

Furthermore, there is significant variation in study designs, with some utilizing exercise stress echocardiography and others employing pharmacologic stress methods. Each approach has unique advantages and limitations, especially in a cardio-oncology context where patient vulnerabilities must be carefully weighed. For example, exercise stress echocardiography, effective in simulating everyday exertion, may not suit all patients, particularly those with severe chemotherapy-induced fatigue or other physical limitations. Conversely, pharmacologic stress echocardiography, more universally applicable, might not provide the same insight into a patient's functional capacity as its exercise counterpart.

The limitations and risks associated with SE in this patient population also merit careful consideration. Technical challenges, such as acquiring high-quality acoustic windows and the need for expert interpretation, present significant obstacles. Additionally, the potential for false positives due to conditions like longstanding hypertension or valvular disease complicates the assessment of cardiac impairment primarily attributable to chemotherapy.

Refining the approach to SE in cardio-oncology necessitates considering both optimal study design and potential confounders. Ideal study designs should include baseline SE parameters before initiating cancer therapy for intra-patient comparisons. Serial imaging at standardized intervals during and after chemotherapy is vital for monitoring progression. Ensuring adequately powered sample sizes with appropriate power analyses, and adjusting for confounders such as age, baseline EF, and comorbidities are essential. Correlating changes observed in SE with cardiac biomarkers and tracking long-term clinical outcomes, particularly heart failure, will provide a more comprehensive understanding of cardiotoxicity.

Addressing potential confounders is equally crucial. For CAD, strategies include excluding subjects with a suspected history of CAD, directly evaluating coronary anatomy, and conducting simultaneous wall motion and perfusion imaging during stress, along with adjusting analyses for CAD risk factors. Distinguishing diastolic dysfunction from cardiomyopathy requires incorporating parameters like mitral inflow patterns, tissue velocities, left atrial volumes, and pulmonary venous flow. Comparisons with age-matched healthy controls can provide further insights.

Regarding the concern of whether a reduction in EF with stress indicates coronary disease or impaired contractile reserve, an optimal design would integrate both wall motion and perfusion imaging during SE. CAD with ischemia typically manifests as stress-induced perfusion defects, as opposed to the global hypokinesia indicative of cardiomyopathy. Reporting achieved peak stress EF, especially when pathologically reduced, can aid in distinguishing between these conditions. Incorporating these comprehensive strategies in study designs will significantly enhance the accuracy and reliability of SE in assessing cardiotoxicity in cardio-oncology patients.

Exercise stress echocardiography is often the first-line stress modality for most patients, including those with cancer, when they are physically able to adequately exercise (51). It allows the evaluation of the heart's ability to respond to increased demands simulating everyday exercise. In cancer patients who are still capable of exercising, post-exertion stress echocardiography can provide us with valuable information. According to a study by Khouri et al. (52), asymptomatic breast cancer patients treated with anthracyclines demonstrated signs of early doxorubicin-induced cardiotoxicity through exercise stress echocardiography, which were undetected at rest. These patients exhibited an impaired capacity to augment LVEF post-peak exercise compared with healthy controls, which suggests a subclinical heart muscle damage from chemotherapy even with normal standard resting measures. The detection of these early changes facilitates intervention prior to progression to heart failure or irreversible cardiomyopathy. The achieved level of exercise can also provide useful prognostic information. As a result, data from exercise stress echocardiography offers significant advantages for both early diagnosis of left ventricular dysfunction and outcome prediction in cancer patients (53).

Dobutamine stress echocardiography (DSE) is a valuable tool for cardiotoxicity detection, particularly regarding the cumulative dose of anthracycline, which helps identify patients at a higher risk of overt cardiotoxicity (47). DSE provides essential insights into the left ventricular contractile reserve (LVCR) in cancer patients. LVCR refers to the capacity of the heart to enhance its pumping function under stress, quantified by the rise in LVEF from rest to peak stress. A decrease in LVCR may indicate impaired myocardial function, even with normal resting LVEF. LVCR is known to predict outcomes in coronary artery disease and is useful for the early identification of cancer patients at risk of late chemotherapy-induced cardiac dysfunction. A previous study utilized DSE in 49 women, pre- and post-anthracycline chemotherapy for breast cancer, and showed that DSE managed to identify patients with reduced LVCR that are susceptible to a significant decrease in LVEF over time. They suggested a threshold of ≥5% drop in LVEF from rest to peak stress to indicate which patients may develop heart failure or impaired pumping function post-treatment (39). Identifying decreased LVCR during chemotherapy can help clinicians implement early interventions, such as modifying drug regimens or reducing the dosage. Moreover, repeated DSE tests post-chemotherapy can reassure recovery in LVCR and cardiac function over time after the end of treatment. For certain drugs like anthracyclines, the risk of late-onset cardiotoxicity may persist for years after therapy ends. Thus, periodic DSE could be utilized as a long-term screening measure to detect late developing dysfunction in specific high-risk survivors, while still evaluating risks versus benefits for each individual case.

Dipyridamole, though less commonly used now, remains an alternative pharmacologic stressor for patients unable to exercise adequately. It works by inhibiting adenosine reuptake, leading to coronary vasodilation and increased cardiac blood flow and oxygen demand. This mechanism is especially relevant during dipyridamole stress echocardiography, where coronary flow reserve (CFR) evaluation of the left anterior descending artery (LAD) can provide insights for patients undergoing cardiotoxic chemotherapy (54, 55). CFR, the ratio of maximum to resting coronary blood flow, reflects the heart’s capability to increase oxygen supply under stress. A CFR lower than 2.0 in the LAD suggests impaired coronary vasodilator capacity, indicative of potential stenosis or microvascular dysfunction (56). In cardio-oncology, dipyridamole stress echocardiography is utilized to detect subclinical cardiotoxicity by observing changes in left ventricular function and wall motion under increased cardiac demands. This method is particularly effective in identifying chemotherapy-induced myocardial damage, which may not be apparent at rest. It has been observed that patients treated with anthracyclines show differences in left ventricular ejection fraction (LVEF) and diastolic function reserve compared to healthy controls. While dipyridamole's pharmacological profile is generally safe, with side effects like headaches, dizziness, or nausea being common and severe reactions rare, its use in contemporary practice has diminished. Nevertheless, it offers a neutral effect on heart rate and contractility, avoiding the potential overestimation of wall motion abnormalities seen with agents like dobutamine (57, 58). Table 3 summarizes the relative advantages and disadvantages of various types of stress echocardiography in cardiotoxicity detection.

**Table 3:** Different types of stress echocardiography in cardiotoxicity detection: advantages and disadvantages.

Type of stress echocardiography	Advantages	Disadvantages
Exercise stress echocardiography	Physiological stress provides the most accurate assessment of cardiac reserve. Low risk and cost if the patient is able to exercise adequately. Detects subclinical LV dysfunction and ischemia.	Limited by the ability and safety of exercise in some patients. Risks of falls, fractures and cardiac events. May miss myocardial fibrosis or subtle diastolic dysfunction.
Dobutamine stress echocardiography	Pharmacologic stress for patients unable to exercise. Detects changes in LV contractile reserve and wall motion abnormalities. Can be used for risk stratification and prediction of late cardiac dysfunction/recovery.	Risks of arrhythmias, hypotension and other side effects. May overestimate dysfunction in patients with valvular disease or hypertension. Anthracyclines/trastuzumab increase dobutamine-associated risks.
Adenosine or dipyridamole stress echocardiography	Non-exercise stress modality with fewer risks and side effects for some patients. Particularly, adenosine is short-acting with few risks of serious reactions. Can assess coronary flow reserve and LV perfusion/function.	Requires IV access and cardiac monitoring as with dobutamine stress. May cause bronchoconstriction or heart block in a small percentage of patients. Diagnostic accuracy may be slightly lower for coronary artery disease detection.

LV, left ventricular; IV, intravenous.

### 2.4 Challenges and limitations of stress echocardiography

Stress echocardiography, a cornerstone in cardiac stress testing, does have some limitations despite its widespread use, yet it is not without its limitations. This paper discusses both the utility and the challenges of SE, particularly in the context of cardio-oncology. Technical complexities, such as the necessity for high-quality acoustic windows and the precision required in serial studies, are significant (59). These factors demand skilled technicians to ensure accurate and reliable results. Moreover, not every patient is a suitable candidate for SE due to specific medical conditions or limitations in adhering to testing protocols (60).

One of the key limitations of SE is its sensitivity in detecting certain types of cardiac damage, such as myocardial fibrosis or early stages of diastolic dysfunction (61). This is of particular concern when monitoring for chemotherapy-induced cardiac damage. Additionally, the risk of false positives, particularly in patients with pre-existing conditions like valvular or coronary artery disease, can lead to challenges in distinguishing between chemotherapy-related cardiac impairment and other causesm (62).

SE, akin to all stress testing modalities, comes with inherent risks. Cancer patients, often dealing with various comorbidities, may face a heightened risk of adverse events during the test. While SE avoids the radiation exposure associated with nuclear perfusion imaging, it is still subject to the risks inherent in exercise or pharmacologic stress, such as falls, arrhythmias, or reactions to contrast media (41).

In conclusion, while SE is an essential tool in the assessment and management of cardio-oncology patients, its limitations and associated risks necessitate careful consideration. Clinicians must weigh these factors against the benefits of SE, ensuring judicious patient selection, thorough monitoring, and comprehensive risk assessment. When applied appropriately, SE can play a significant role in effective cardiac monitoring and risk stratification for cancer patients undergoing treatment.

### 2.5 Future direction

Speckle tracking echocardiography, also known as strain imaging, detects subtle changes in the motion and deformation of the heart muscle, by utilizing ultrasound speckles in the myocardial wall rather than relying on endocardial borders. Speckle tracking can reveal signs of cardiotoxicity even with normal LVEF. Previous studies have shown reduced global longitudinal strain in chemotherapy patients despite preserved LVEF (63). This earlier detection of dysfunction could allow therapeutic modifications before the occurrence of significant damage. A more comprehensive evaluation of the left ventricular structure and function can be acquired through 3D echocardiography. Unlike 2D imaging, it provides a complete view of the ventricle, not just selected slices. It has been shown that 3D stress echocardiography is useful in detecting subtle changes resulting from cardiotoxic drugs that could otherwise go unnoticed (64). This method could become an essential tool for monitoring high-risk patients, where even a minor decrease in the function needs to be addressed. Contrast echocardiography improves the assessment of hard-to-visualize left ventricular wall segments through ultrasound contrast agents, or gas-filled microbubbles. The contrast helps to define endocardial borders, which is particularly beneficial during stress echocardiography, where images can be challenging to capture. Studies have shown that contrast echocardiography during stress provides more comprehensive data and higher confidence compared with non-contrast stress echocardiography (65, 66). This allows for earlier or more reliable detection of cardiotoxicity.

Cardiac magnetic resonance imaging (CMR) emerged as another technique for monitoring cardiac function during chemotherapy. It can deliver high-definition images of the myocardium and chambers, boasting some advantages over ultrasound in certain circumstances. In combination with stress echocardiography, CMR has been studied for a comprehensive evaluation of the structure and stress-induced changes in the wall motion (67). Integrating these two modalities could provide substantial advantages for the detection and monitoring of cardiotoxicity resulting from various treatments.

With the advancement in chemotherapy and other cardiotoxic treatments, long-term heart health should be adequately addressed. Stress echocardiography will likely play an expanding role in risk stratification, early detection of cardiotoxicity and guiding treatment modifications to prevent long-term heart failure or other complications. Emerging echocardiography technologies may eventually allow for the early detection of the smallest changes indicating cardiotoxicity. Other modalities, like cardiac MRI, might be used alongside stress echocardiography for a comprehensive monitoring. Emerging biomarkers for cardiotoxicity detection, combined with imaging, could potentially enable individually tailored treatment plans based on a patient’s risk profile and response to drugs. The overall goal would be to pre-identify a patient's sensitivity to the cardiotoxic effects of a medication, so that the frequency of monitoring and specific types of medications can be proactively tailored to minimize cardiac issues while also achieving the treatment goals.

## 3 Conclusion

In summary, SE may play a role in the management of cardiotoxicity. However, further research is essential to establish its optimal use in clinical practice.
